# The Infection Biology of *Sphaerulina musiva*: Clues to Understanding a Forest Pathogen

**DOI:** 10.1371/journal.pone.0103477

**Published:** 2014-07-29

**Authors:** Ruqian Qin, Jared M. LeBoldus

**Affiliations:** Department of Plant Pathology, North Dakota State University, Fargo, North Dakota, United States of America; USDA Forest Service - RMRS, United States of America

## Abstract

Trees in the genus *Populus* and their interspecific hybrids are used across North America for fiber production and as a potential source of biofuel. Plantations of these species are severely impacted by a fungal pathogen, *Sphaerulina musiva*, the cause of leaf spot and stem canker. An inoculation protocol that does not rely on stem wounding to achieve infection was recently developed. Using this protocol two experiments were conducted to examine infection biology and disease development in the *S. musiva*-*Populus* interaction. In the first experiment non-wounded stems of one moderately resistant clone (NM6) and one susceptible clone (NC11505) were inoculated and examined by scanning electron microscopy at six different times (6 h, 12 h, 24 h, 72 h, 1 week, and 3 weeks) post-inoculation. The images indicate that the pathogen appears to enter host tissue through small openings and lenticels and that there are no significant differences in the penetration rate between the moderately resistant (NM6) and susceptible (NC11505) clones at 12 h post-inoculation. In a second experiment a histological comparison of stem cankers for resistant clone DN74 and susceptible clone NC11505 were conducted at three time points (3 weeks, 5 weeks, and 7 weeks) post-inoculation. Distinct differences in disease development were apparent between the resistant and susceptible clones at each time point, with the susceptible clone exhibiting a weak and delayed defense response. These results suggest, that following penetration, the pathogen may be able to interfere with the defense response in the susceptible host.

## Introduction

Poplar trees, as foundation species [1] within many ecosystems, occur across most of North America in native populations hundreds- to thousands-of-years old. These species are ecologically and commercially important as a result of their broad geographic distribution and potential use as a bioenergy feedstock. The primary limitation to the use of *Populus* spp. for fiber, biomass, and bioenergy in central and eastern North America are the fungal diseases, Septoria leaf spot and stem canker, caused by *Sphaerulina musiva* (Peck) Verkeley, Quaedvlieg, & Crous (syn. *Septoria musiva* Peck; Teleomorph = *Mycosphaerella populorum* Thompson) [2]–[4]. Severe leaf-spot disease can result in premature defoliation and stem and branch infections can lead to defects, breakage, and mortality [2]–[5]. Septoria canker has the greatest impact in pulp and bioenergy plantations due to the potential for a single infection to kill a tree [7]. Planting resistant genotypes (clones) is typically considered the most effective way to manage this disease [3], [4]. As a result, a large number of studies have been conducted in both the field [5]–[7] and greenhouse [8]–[10] in order to identify disease resistant clones. Although some progress has been made, the *S. musiva*-*Populus* interaction is still poorly understood.

One of the major barriers to understanding this interaction has been uncertainty regarding the mode of entry of the pathogen into stem tissue. Without an understanding of the pathogen’s infection biology it is difficult to elucidate resistance mechanisms. The literature indicates that *S. musiva* is typically considered to be a pathogen associated with wounds [2]–[5], [11]. This resulted in the widespread use of a wound inoculation protocol in order to incite disease and compare relative levels of resistance [7]–[9]. However, several authors have also indicated that the pathogen may penetrate host tissue through natural openings [2], [4], [10], [12]. For example, Bier [4] reported necrotic lesion development surrounding lenticels, petioles, and stipule scars of non-wounded hybrid poplars. On highly susceptible clones *S. musiva* appears to infect stems directly without any association with natural openings [12]. To date it has not been possible to examine the infection biology of this pathogen due to the relatively low infection frequency reported in these studies [10], [11]. The development of a consistent non-wound inoculation protocol [13] has eliminated this constraint. This has allowed for the first time, a detailed microscopic description of the *S. musiva*-*Populus* interaction in the absence of wounding.

The majority of studies examining the infection biology of canker fungi rely on artificial wounding and large amounts of inoculum, grown on artificial media, to incite disease [14]. These studies are typified by the formation of a necrophylactic periderm (NP) in response to wound inoculation [15]–[19]. This is consistent with the only study examining stem responses of hybrid poplars wound inoculated with *S. musiva* [20]. In that study the authors compared the reactions of susceptible clone NC11505 (*Populus maximowiczii* A. Henry×*Populus trichocarpa* Torr. & A. Gary) and resistant clone DN34 (*Populus deltoides* Marsh×*Populus nigra* L.) 7 weeks post-inoculation (PI) [20]. Differences between the clones were observed in terms of the position, thickness, and continuity of the NP layer. In the resistant clone DN34, a thick continuous layer of NP developed in close proximity to the inoculation point potentially limiting pathogen development [20]. In contrast, the susceptible clone NC11505 developed extensive necrosis with several successive layers of thin NP, located further away from the point of inoculation [20]. This NP was frequently disrupted by phloem fibers [20]. NP disruption was absent in the resistant clone and the authors hypothesized that the pathogen was able to circumvent the NP by passing through these phloem fibers [20].

Disease resistance mechanisms, both structural and biochemical, act against a pathogen prior to and following penetration [21]. Pre-penetration resistance reported in other pathosystems includes structural differences in the infection courts of host genotypes [22] and rapid pathogen recognition and subsequent activation of disease resistance pathways [23]. The differences among hybrid poplar clones in terms of the number of lesions per cm^−1^ [13] suggests that pre-penetration resistance may be important in the *S. musiva-Populus* interaction. For example, infection frequency may be correlated with the number of lenticels and other natural openings present on the stem.

Post-penetration resistance includes anatomical and/or biochemical barriers that physically and/or chemically exclude the pathogen [24]. Several authors have suggested that NP formation, similar to that described above [20], may be a determining factor in resistance to fungal invasion by acting in this manner [15]–[17]. However, NP is considered to be a non-specific host response resulting from disruption of the phellogen (cork cambium) [18], [19], [25]. This divergence of opinion can largely be attributed to the necessity of wounding in order to incite disease in the majority of the tree-pathogen interactions studied to date [14]. Examining the role of NP formation following inoculation with a spore suspension of *S. musiva*, will begin to disentangle host response to wounding from host response to pathogen invasion. These results will potentially provide new insights into disease resistance in forest trees.

This study is the first detailed microscopic description of the infection biology of the *S. musiva-Populus* interaction in the absence of wounding. The responses of a resistant clone (DN74 = *P. deltoides*×*P. nigra*), a moderately resistant clone (NM6 = *P. maximowiczii*×*P. nigra*), and a susceptible clone (NC11505 = *P. maximowiczii*×*P. trichocarpa*) were compared in two separate experiments. In the first experiment scanning electron microscopy (SEM) was used to compare the mode of infection between the moderately resistant (NM6) and susceptible (NC11505) clones at 6 time points post-inoculation (PI; 6 h, 12 h, 24 h, 72 h, 1 week, and 3 weeks). In a second experiment the histological responses, in the absence of wounding, of a resistant (DN74) and a susceptible (NC11505) clone were compared at three time points PI (3 weeks, 5 weeks, and 7 weeks). The overall aim of these two experiments was to characterize the infection process and disease development of hybrid poplar stems inoculated with *S. musiva* in the absence of wounding. The specific objectives were to: (1) determine the infection court(s) used by the pathogen; (2) examine the role of NP in disease resistance; and (3) compare clones with different levels of susceptibility in terms of pre- and post-penetration resistance.

## Materials and Methods

### Infection biology

#### Host plant propagation

Dormant branches of the susceptible clone NC11505, moderately resistant clone NM6, and the resistant clone DN74 were collected in February, 2012 at the University of Wisconsin-Madison Arlington Agricultural Experiment Station (43°18′7.06 N Lat.; 89°20′47.98 W Long.), and cut into 10-cm lengths. No specific permission was required for the collection of this plant material. Cuttings were initially soaked in distilled water at room temperature (21°C) for 48 h, and then planted in SC10 Super cone-tainers (Stuewe & Sons Deepots D40 cell; Stuewe & Sons Inc., Tangent, OR) containing SunGro growing medium (SunGro Professional Mix #8; SunGro Horticulture Ltd., Agawam, MA) amended with 12 g of nutricote slow-release fertilizer (15-9-12) (N-P-K) (7.0% NH_3_-N, 8.0% NO_3_-N, 9.0% P_2_O_5_, 12.0% K_2_O, 1.0% Mg, 2.3% S, 0.02% B, 0.05% Cu, 0.45% Fe, 0.23% chelated Fe, 0.06% Mn, 0.02% Mo, 0.05% Zn; Scotts Osmocote Plus; Scotts Company Ltd., Marysville, OH). This was supplemented on a weekly basis with a 500 ppm solution of liquid fertilizer (20-20-20) (N-P-K) (3.94% NH_3_-N, 6.05% NO_3_-N, 10.01% CO(NH_2_)_2_, 20.0% P_2_O_5_, 20.0% K_2_O, 0.05% Mg, 0.0068% B, 0.0036% Cu, 0.05% Chelated Fe, 0.25% Mn, 0.0009% Mo, 0.0025% Zn; Scotts Peters Professional; Scotts Company Ltd., Marysville, OH). Planted cuttings were placed on a greenhouse bench with an 18-h photoperiod, supplemented with 600W high-pressure sodium lamps, and a 20°C/16°C (day/night) temperature regime. Trees were transplanted into plastic pots (22-cm-deep×22.5-cm-diameter; Stuewe & Sons Treepot CP59R: Stuewe & Sons Inc., Tangent, OR) when they reached a height of 30 cm.

#### Pathogen propagation and inoculation

*Sphaerulina musiva* was isolated from branch cankers, collected from hybrid poplars located at VERSO Paper company’s tree nursery near Belle River, MN (45°59′44.00 N Lat.; 95°12′50.91 W Long.). This nursery is on private land and permission was obtained from VERSO Paper Company to do the collections. The cankers were first soaked in a 5% bleach solution (NaClO 6%; Homelife Bleach Regular Scent; KIK Custom Products Inc., Houston, TX) for 2 minutes and then rinsed twice with sterile-distilled water. Bark was carefully removed from the canker margin and 4-mm-long slivers of tissue were placed on V-8 juice agar (137 ml V-8 juice, Campbell Soup Company, Camden, NJ; 1.5 g CaCO_3_, ReagentPlus, Research Organics Inc., Cleveland, OH; 15.2 g agar Difco, Franklin Lakes, NJ and 625 ml de-ionized water). Petri plates were sealed with Parafilm and incubated at room temperature (21°C) 30 cm below continuous light (Gro-Lux full spectrum fluorescent bulbs: Sylvania; Osram Gmbh, Munich, Germany). After 1 week, colonies resembling *S. musiva* were transferred onto a second V-8 juice agar plate and identified based on conidial morphology [26]. Species identity was subsequently confirmed by multi-locus genotyping (LeBoldus *et al.* unpublished data). Conidia had 2 to 4 septations and ranged in size from 28–54×3.5–4 µm. Pure *S. musiva* cultures were stored at −80°C in vials containing 1 ml of 50% glycerol solution.

Each isolate (MN7, MN11, and MN23) was recovered from cold storage by pouring 1 ml of the glycerol solution onto one Petri plate containing V-8 juice agar. Three plates of each isolate were grown on the light bench described above. Five days later, sporulating colonies were aseptically transferred onto 13 new V-8 juice agar plates and incubated on the light bench until sporulation occurred. Conidia were harvested by flooding the plates with 5 ml of sterile-distilled water and lightly rubbing the surface of the plate with a sterile loop. For each isolate, conidial suspensions harvested from each plate were combined and the concentration was adjusted to 1×10^6^ conidia ml^−1^. Equal volumes of each isolate were combined and the bulked spore suspension was used for inoculation. Four weeks after transplanting, the stems of each tree were inoculated with a spray bottle [13]. Twelve trees of the susceptible clone (NC11505) and twelve trees of the moderately resistant clone (NM6) were inoculated. In addition, two control trees from each clone were mock-inoculated in an identical manner, except that sterile distilled water was used rather than a spore suspension.

#### Experimental design

The experimental design was a completely randomized design. At each of six time points (6 h, 12 h, 24 h, 72 h, 1 week, and 3 weeks) PI stem segments from two trees of each clone were harvested, with one exception. Only a single tree was harvested at 1 week. At time points where symptoms were not visible (6 h, 12 h, 24 h, 72 h, 1 week), three randomly selected 5-cm segments were harvested from each tree. At 3 weeks PI, when symptoms were visible, two cankers were randomly selected from the lower 15 cm of each stem. Cankers were sampled such that a 5-cm segment of stem, centered on the canker, was collected. In addition, a single mock-inoculated control tree of each clone was harvested at 3 weeks PI and three, 5-cm segments, from the lower 15 cm and the upper 15 cm of the tree, were harvested. All samples were fixed in a 2.5% solution of gluteraldehyde in 0.2 M sodium phosphate buffer (pH 7.4; Tousimis Research Corporation, Rockville, MD) and stored at 4°C for three days. A total of four canker segments and eight control segments per time point were examined for each clone.

#### Scanning electron microscopy

The fixed stems were split longitudinally, so that both surfaces could be observed, and then dehydrated in an ethanol series from 30% to 100%. The split samples were critical-point dried using an Autosamdri 810 critical-point drier (Tousimis Research Corporation, Rockville, MD) with liquid carbon dioxide as the transitional fluid. Longitudinal sections were attached to aluminum mounts with silver paint (SPI Supplies, West Chester, PA) and sputter coated with gold/palladium (Balzers SCD 030, Balzers Union Ltd., Liechtenstein). Images were obtained using a JEOL JSM-6490LV SEM (JEOL Ltd., Japan) operating at an accelerating voltage of 15 kV. To estimate the number of germ tubes that had entered natural openings, images at 12 h PI were used. A total of 100 spores were counted in 2 to 3 fields of view. Subsequently, a two-sample t-test (df = 1; *α* = 0.05) was conducted to compare the penetration rate of the fungus into the moderately resistant (NM6) and susceptible (NC11505) clones.

### Host response to non-wound inoculations

#### Host plant propagation, pathogen propagation, and inoculation

Plants were propagated in a similar manner to that described above, with the following exception: DN74 rather than NM6 was used. It was necessary to substitute NM6 with DN74 as there were insufficient trees of NM6 to conduct the experiment. Inoculum production and inoculation were conducted as described above.

#### Experimental design

The experimental design was a completely randomized design. A total of six stem segments, approximately 5 cm in length and centered on cankers, were collected from each clone at each of the following time points: 3 weeks, 5 weeks, and 7 weeks PI. Six stem segments from a mock-inoculated control of each clone were also harvested at 7 weeks PI. In total 48 segments (36 inoculated and 12 controls) were fixed in 10 ml of a formalin-acetic acid-alcohol (FAA, 10∶5∶50) solution for one week at 21°C.

#### Histology

Fixed stem segments were dehydrated in an automated tissue processor (Leica Microsystems Inc., Buffalo Grove, IL) following the manufacturer’s instructions. The samples were then embedded with Paraffin Plus (Fisher Scientific Co., Houston, TX) using a Leica embedding machine (Leica Microsystems Inc., Buffalo Grove, IL). Longitudinal and transverse sections of the cankers were made using a rotary microtome (Leica Microsystems Inc., Buffalo Grove, IL) set to a thickness of 20 µm. Three segments were sectioned transversely through the top, middle, and bottom of each canker and the remaining three were sectioned longitudinally through the center of each canker. Several sections were made at each location. All sections were placed on microscope slides (Fisher Scientific Co., Houston, TX), de-waxed in Histo-Clear (Fisher Scientific Co., Houston, TX), stained using a Safranin-O Fast-green protocol [27], and mounted using Permount mounting medium (Sigma-Aldrich Co., St. Louis, MO). Sections were examined by light and fluorescence microscopy using a Zeiss Axio Imager M2 microscope. Blue auto-fluorescence viewed with ultraviolet light (Excitation filter G 365, Beam Splitter FT 395, Emission filter BP 445/50) and green auto-fluorescence viewed with blue-green light (Excitation filter BP 450–490, Beam Splitter FT 510, Emission filter BP 515–565) were used to visualize host responses.

## Results

In both experiments necrotic lesions were first observed on the susceptible clone (NC11505) at 2 weeks PI and on the moderately resistant (NM6) and resistant clone (DN74) at 3 weeks PI. Initially, lesions appeared as areas of water-soaked cells on the surface of the stem. The majority of lesions developed on the lower 15 cm section of inoculated trees and were rarely observed on the upper 15 cm section. Disease development differed between resistant and susceptible clones. Water-soaked areas on the resistant clone (DN74) and moderately resistant clone (NM6) developed swollen margins. Seven weeks PI any necrosis that developed on these clones was completely contained by these swollen margins. In the case of the susceptible clone (NC11505), the water-soaked areas became necrotic with a dark brown to black appearance. Three weeks PI necrotic lesions developed tan centers with pycnidia oozing pinkish spore tendrils. At 7 weeks PI necrotic lesions had coalesced completely girdling the stem of the susceptible clone (NC11505). No symptoms developed on control trees in either experiment.

### Infection biology

**Figure 1:**
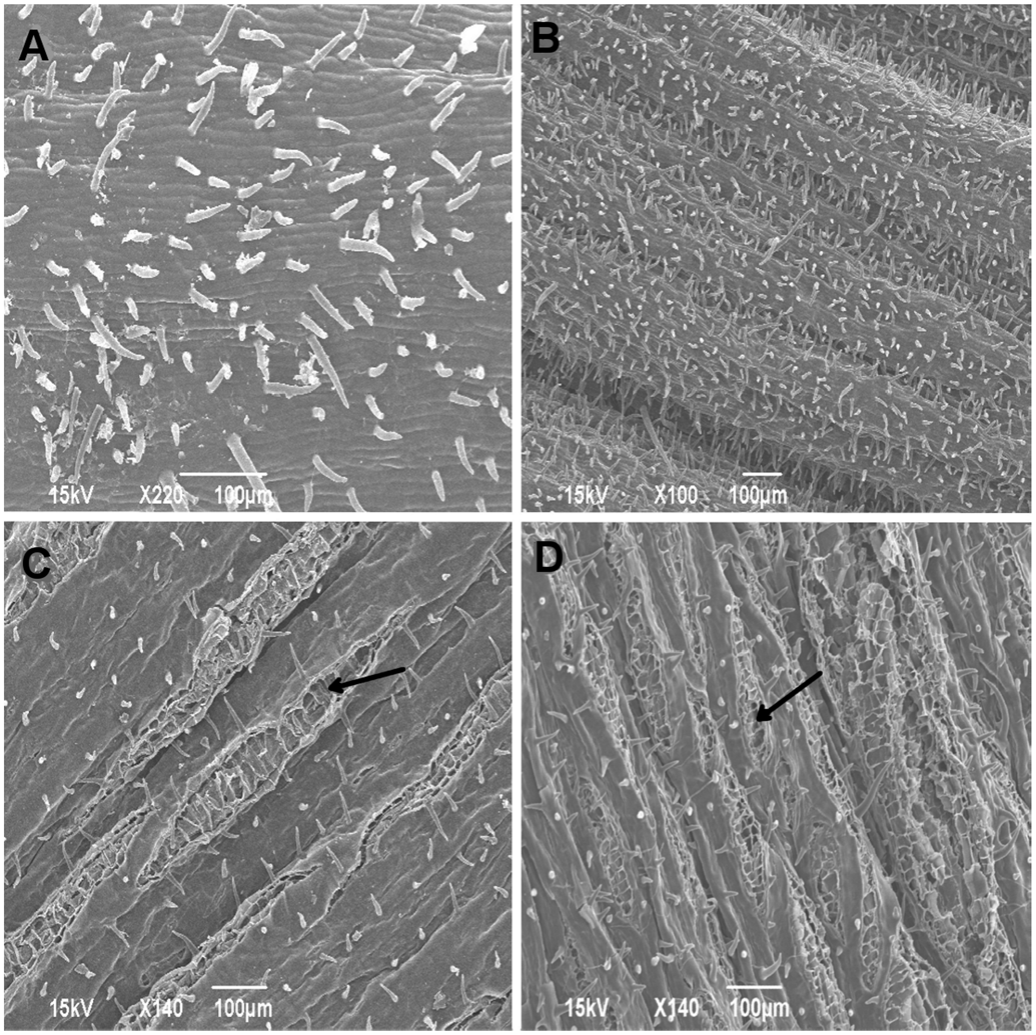
Scanning electron micrographs (SEM) of the surface of mock-inoculated control trees at two different heights post-inoculation (PI). (A) Surface of upper 15 cm stem segment, lacking any openings/lenticels, collected from the susceptible clone NC11505. (B) Surface of upper 15 cm stem segment, lacking any openings/lenticels, collected from the moderately resistant clone NM6. (C) Surface of lower 15 cm stem segment, with small openings/lenticels indicated by the arrow, of clone NC11505. (D) Surface of lower 15 cm stem segment, with small openings/lenticels indicated by an arrow, of clone NM6. Magnification and scale bars included on the bottom of each image.

**Figure 2:**
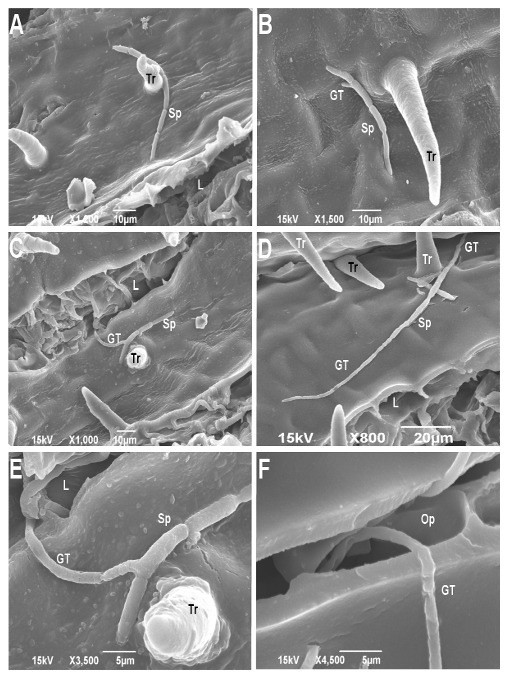
Scanning electron micrographs (SEM) of stems inoculated with a conidial suspension of *Sphaerulina musiva* at two different times (6 h and 12 h) post-inoculation (PI) depicting germination and penetration on the moderately resistant (NM6) and susceptible (NC11505) clones. (A) SEM micrograph of stem surface of NC11505 depicting *S. musiva* conidium 6 h PI. (B) SEM micrograph of stem surface of NM6 and conidium of *S. musiva* 6 h PI. (C) SEM micrograph of stem surface of NC11505 with conidium and germ tube of *S. musiva* entering a lenticel 12 h PI. (D) SEM micrograph of stem surface of NM6 with conidium and germ tube of *S. musiva* penetrating a small opening 12 h PI. (E) Micrograph (C) at increased magnification depicting penetration of lenticel by a germ tube. (F) SEM micrograph of stem surface of resistant clone NM6 with germ tube of *S. musiva* penetrating a small opening 12 h PI. Tr = trichome, Sp = conidium, L = lenticel, GT = germ tube, Op = small opening. Magnification and scale bars included on the bottom of each image.

An examination of the upper 15 cm and lower 15 cm of the stem revealed the lack of small openings on the upper 15 cm sections [Figure 1]. This observation was consistent for both the moderately resistant (NM6) and susceptible (NC11505) clones [Figure 1]. At 6 h PI, spores had adhered to the stem surface and had begun to germinate on both clones [Figure 2]. At 12 h PI, germ tubes appeared to have entered host tissue through either lenticels or small openings on both resistant and susceptible clones [Figure 2]. At no time were infection structures, such as appressoria, visible. Germ tubes did not grow towards the nearest opening but appeared to meander across the surface of the inoculated stem, entering openings at random [Figure 2]. The images at 72 h PI, 1 week PI, and 3 weeks PI were similar for both the moderately resistant and susceptible clones [Figure 2].

A two-sample t-test comparing the mean number of germ tubes appearing to have penetrated host tissue indicated no significant difference (*P* = 0.41) between the moderately resistant (NM6; 35.75%) and susceptible (NC11505; 42.75%) clones [Table 1].

**Table 1:** A comparison of the mean, range, and standard deviation of penetration rates on the moderately resistant clone NM6 (*Populus maximowiczii*×*P. nigra*) and susceptible clone NC11505 (*P. maximowiczii*×*P. trichocarpa*) inoculated with a spore suspension of *Sphaerulina musiva*.

	**Percentage of germ tubes entering natural openings (%)**		
**Clone**	**Mean**	**Range**	**Std. deviation**
NC11505	42.75 (n = 2) a	35–55	8.80
NM6	35.75 (n = 2) a	28–45	8.10

The percentage of germ tubes entering natural openings was estimated by counting a total of 100 spores in two to three fields of view for each tree using a scanning electron microscope. Means were compared using a two-sample t-test (degrees of freedom = 1) means with the same letter are not significantly different (*P* = 0.41).

### Host response to non-wound inoculations

#### Histology of control plants

**Figure 3:**
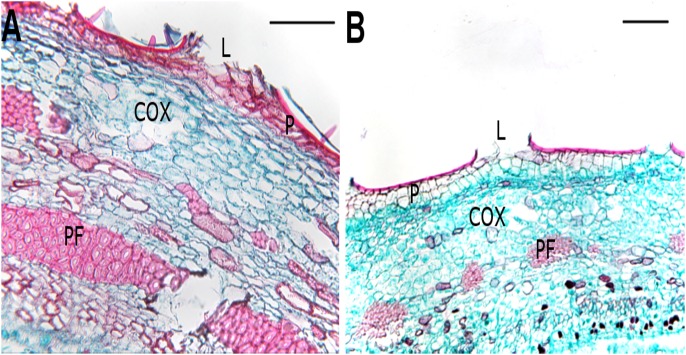
Bright field micrographs of transverse sections through 7-week-old mock-inoculated controls of the resistant (DN74) and susceptible (NC11505) clones depicting the gross anatomy of stem tissue. (A) Stem anatomy of the mock-inoculated clone DN74. (B) Stem anatomy of mock inoculated clone NC11505. COX = Cortex, L = Lenticel, P = Periderm, PF = Phloem fiber. Scale bars = 200 µm.

The anatomy of the mock inoculated controls DN74 and NC11505 resembled the descriptions of DN34 and NC11505 made by Weiland and Stanosz [25]. The transverse sections can be subdivided into three layers (periderm, cortex, and xylem) containing primary phloem fibers, phloem, vascular cambium, and xylem vessels [Figure 3]. The epidermis was typically 1 to 2 cell layers thick and appeared blue-green under fluorescent light [Figure 3]. Lenticels were visible throughout the epidermis and developing periderm [Figure 3]. Cortex was located adjacent to the periderm beyond the phloem tissue. Occasionally, phloem fibers with thick cell walls, were located within the cortex and appeared bright purple under fluorescent light.

#### Histology 3 weeks post-inoculation

**Figure 4:**
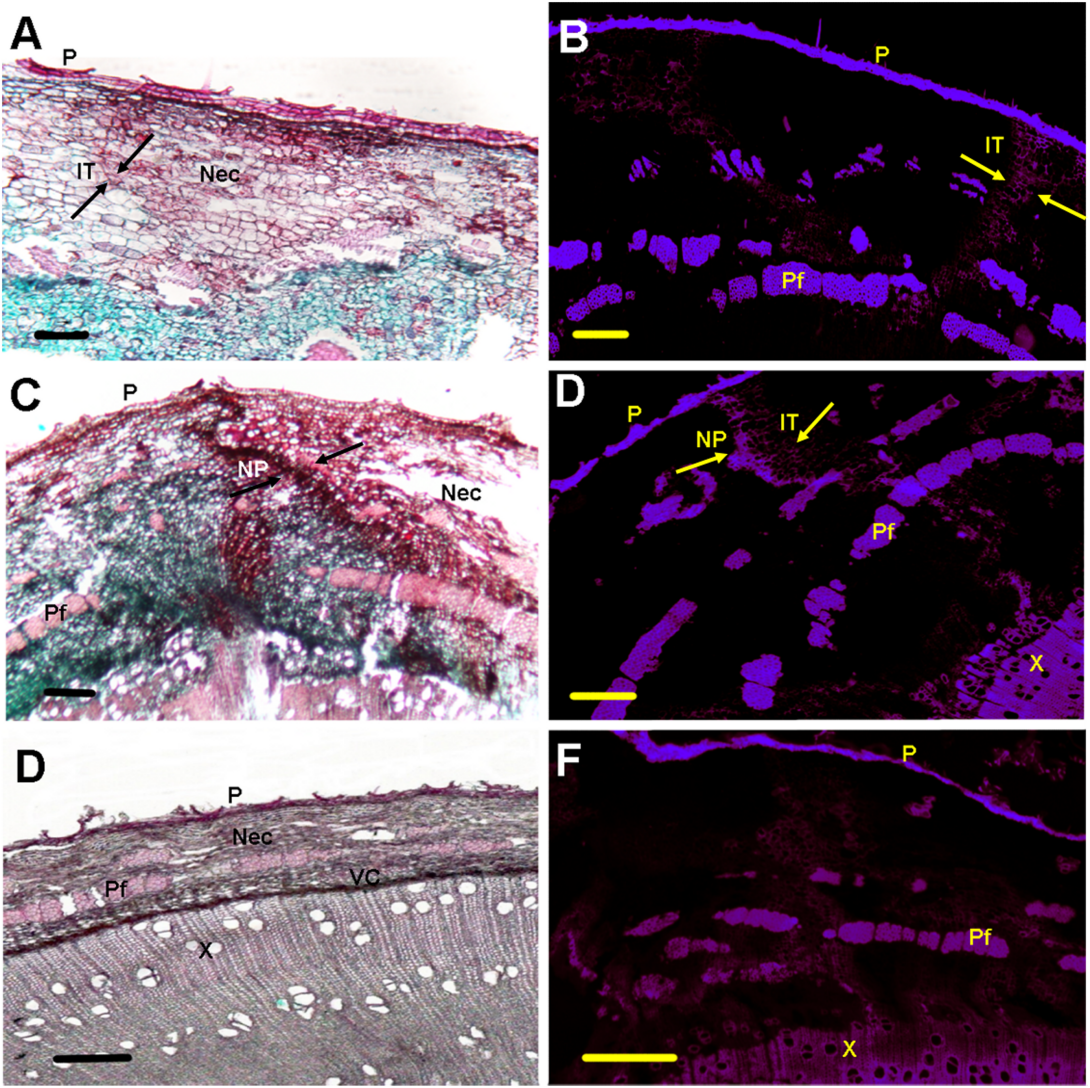
Transverse sections of susceptible clone NC11505 at 3 time points (3 weeks, 5 weeks, and 7 weeks) post-inoculation (PI) depicting anatomical responses to inoculation with a conidial suspension of *Sphaerulina musiva*. (A) Bright field micrograph of necrotic stem lesion 3 weeks PI. Necrotic area (Nec) bounded by layer of impervious tissue (IT indicated with arrow). (B) Fluorescent micrograph of necrotic lesion 3 weeks PI. IT layer visible as light purple fluorescence (indicated with arrows). (C) Bright field micrograph of necrotic lesion 5 weeks PI. Nec is bounded by a layer of necrophylactic periderm (NP; indicated with arrow). (D) Fluorescent micrograph of necrotic lesion 5 weeks PI. Nec is bounded by IT layer and NP layer (indicated with arrows). (E) Bright field micrograph of necrotic stem 7 weeks PI. Entire cortex (COX) is necrotic and filled with collapsed cells. (F) Fluorescent micrograph of necrotic lesion 7 weeks PI. NP and IT are absent from the Nec. Blue auto-fluorescence viewed under ultraviolet light. Filter parameters: Excitation filter G 365, Beam Splitter FT 395, Emission filter BP 445/50. Green auto-fluorescence viewed under ultraviolet light. Filter parameters: Excitation filter BP 450–490, Beam Splitter FT 510, Emission filter BP 515–565. P = periderm. Pf = primary phloem fiber. X = Xylem. VC = Vascular cambium. Scale bars = 200 µm.

*Susceptible clone NC11505.* Symptomatology was similar to that described previously for the susceptible clone. Transverse sections through the midpoint of each canker revealed light-brown necrosis of the vascular cambium. Both fluorescent and light micrographs of transverse sections indicated the presence of an impervious tissue (IT) layer [Figure 4], which was chromophilic and amorphous under ultraviolet light. Hyphae were clearly visible in the cortex 3 weeks PI [Figure 4]. No evidence of NP formation was observed.

**Figure 5:**
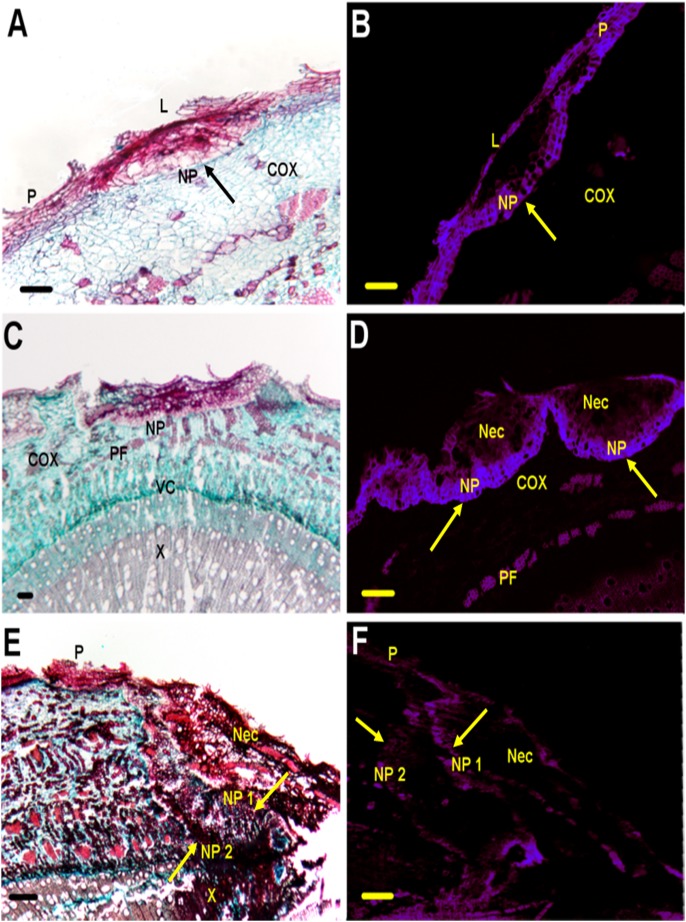
Transverse sections of resistant clone DN74 at 3 time points (3 weeks, 5 weeks, and 7 weeks) post-inoculation (PI) depicting anatomical responses to inoculation with a conidial suspension of *Sphaerulina musiva*. (A) Bright field micrograph of necrotic lenticel (L) 3 weeks PI. Necrophylactic periderm (NP; indicated by an arrow) is apparent immediately below the infected L at the margin between the cortex (COX) and Periderm (P). (B) Fluorescent micrograph of necrotic lenticel 3 weeks PI. NP layer is evident as thick square shaped cells fluorescing bright purple immediately below the lenticel within the COX (indicated by an arrow). (C) Bright field micrograph of necrotic lesion, bounded by NP, 5 weeks PI. (D) Fluorescent micrograph of two necrotic lesions 5 weeks PI. NP is evident as thick layer of purple fluorescing cells (indicated by arrows) immediately below the necrotic area (Nec). (E) Bright field micrograph of Nec 7 weeks PI. Nec is bounded by two successive layers of NP (indicated by arrows), with occluded xylem (X) cells (dark red to black cells in the X) adjacent to the Nec. (F) Fluorescent micrograph of necrotic lesion with two successive layers of NP appearing purple (indicated by arrows). Blue auto-fluorescence viewed under ultraviolet light. Filter parameters: Excitation filter G 365, Beam Splitter FT 395, Emission filter BP 445/50. Green auto-fluorescence viewed under ultraviolet light. Filter parameters: Excitation filter BP 450–490, Beam Splitter FT 510, Emission filter BP 515–565. P = periderm. Pf = Primary phloem fiber. VC = Vascular Cambium. Scale bars = 200 µm.

*Resistant clone DN74*. Disease development was similar to that described for NM6. Swelling developed along the margin of necrotic tissue. Fungal invasion appeared to be restricted to lenticels and adjacent cortex by the rapid formation of NP visible in transverse sections [Figure 5]. Hyphae were not observed in the cortex of the resistant clone.

#### Histology 5 weeks post-inoculation

**Figure 6:**
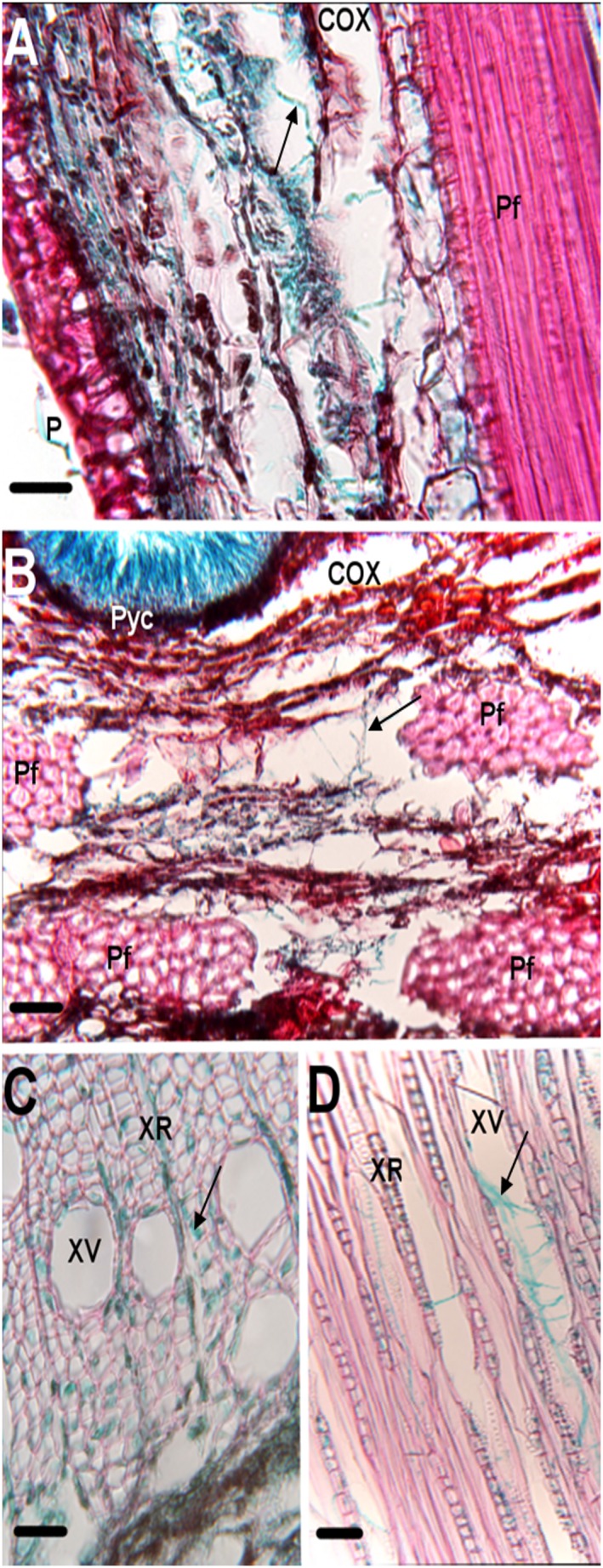
Bright field micrographs of susceptible clone (NC11505), at three different time points (3 weeks, 5 weeks, and 7 weeks) post-inoculation (PI) with blue stained hyphae, indicated by arrows, visible in different tissues. (A) Longitudinal section depicting hyphal growth in cortex at 3 weeks PI. (B) Transverse section through cortex depicting hyphal growth at 5 weeks PI. (C) Transverse section through xylem at 7 weeks PI depicting hyphal growth in xylem vessels (XV) and rays (XR). (D) Longitudinal section through xylem tissue depicting hyphal growth in vessels and rays. COX = Cortex, Pf = Primary phloem fiber, Pyc = Pycnidium, P = Periderm. Scale bars = 200 µm.

*Susceptible clone NC11505.* After 5 weeks PI, necrotic lesions had enlarged longitudinally and cankers had begun to coalesce. Without magnification, transverse sections of cankers were observed to have yellowish-brown staining of the xylem tissue and pycnidia were observed forming at the stem surface. Several cankers had developed NP at an oblique angle extending from the periderm to the xylem [Figure 4]. The NP appeared discontinuous under fluorescent light and was interrupted at several locations by phloem fibers [Figure 4]. Hyphae were present in the periderm and cortex adjacent to the vascular cambium [Figure 6].

*Resistant clone DN74*. The swollen margins of the canker appeared to have completely contained the small necrotic area. Under fluorescent light the NP layer was visible in the cortex in close proximity to the epidermis, restricting necrosis from the vascular cambium [Figure 5].

#### Histology 7 weeks post-inoculation

*Susceptible clone NC11505.* Coalesced cankers had girdled the stem at multiple locations. The NP layer was rarely visible at this time point, fluorescing weakly under UV light [Figure 4]. All cells associated with necrotic tissue had collapsed [Figure 4] and hyphae were visible throughout the periderm, cortex, and xylem [Figure 6].

*Resistant clone DN74*. Macroscopically, all cankers examined at 7 weeks PI were similar in appearance to those at 5 weeks PI. The necrotic area was contained by the NP and no further disease development had occurred. However, at the microscopic level several cankers (four out of six segments sampled) had developed necrosis from the periderm to the vascular cambium and xylem. In these cases two successive layers of NP were evident [Figure 5], the first appearing to be incomplete, extending into the cortex, and the second complete, extending from the periderm to the xylem [Figure 5]. Xylem cells in close proximity to the NPs were occluded and the vascular cambium appeared to be regenerating xylem tissue [Figure 5]. No hyphae were visible in longitudinal sections of the xylem.

## Discussion

*Sphaerulina musiva* is frequently reported in the literature to cause cankers in association with wounded stems and branches [2]. However, several studies where researchers inoculated non-wounded stems of *Populus* spp. with *S. musiva* have reported the development of necrotic lesions [4], [10]–[12]. In these studies cankers have typically developed at stipule scars [11], lenticels [4], [10]–[12], the base of leaves [10], and on petioles [4], [10]. Disease development following inoculation is similar across all studies. Initially, small water-soaked lesions appeared on stems of inoculated trees 2 to 3 weeks PI then these lesions rapidly became necrotic. On the susceptible clones, necrotic lesions coalesced with no visible macroscopic host response, eventually girdling the tree. On resistant clones, the margins of the necrotic lesions became swollen as the tree responded to infection. Disease progress was similar to what has been previously reported in terms of both the location of canker development and the responses of the resistant (DN74), moderately resistant (NM6), and susceptible (NC11505) clones.

Disease development was similar between NM6 and NC11505 in the early stages of infection. At 12 h PI there was no significant difference between the moderately resistant and susceptible clones in terms of the number of spores that had found a lenticel or small opening to enter [Figure 2], [Table 1]. There was no evidence of direct penetration or the formation of infection structures on either clone. A similar mode of infection has been reported for *Quambalaria* spp. causing leaf and shoot blight of *Eucalyptus* spp. which penetrates via stomata and small wounds [28]. A second similarity between *S. musiva* and *Quambalaria* spp. is the haphazard pattern of growth. The germ tubes of *S. musiva* would grow over nearby infection courts (stomata/lenticel/wound) and penetrate an infection court further away [28]. The literature suggests that this pattern of growth may be related to a chemotrophic rather than a thigmotrophic mechanism of attraction [29], [30].

In this inoculation experiment and in others conducted by our research group, the majority of cankers appear to develop on the lower 15 cm segment of the inoculated trees [13], [31]. One possible explanation of this phenomenon may be the developmental stage of the host. The youngest tissue, at the top of the tree, is covered by a thin epidermis lacking natural openings for penetration of *S. musiva* [Figure 1]. As the periderm and lenticels begin to form the epidermis stretches and then splits, the resulting lenticels and small openings provide potential infection courts [Figure 2]. A second possibility is the movement of spores along with dripping water following inoculation. Although the trees were uniformly sprayed at the time of inoculation it is possible that water dripping from the top of the tree may carry spores with it, resulting in more infections on the lower portion of the stem. Given that the pathogen appears to penetrate predominantly through lenticels and other small openings, there may be differences in lenticel structure among clones which could be exploited in the search for Septoria canker resistance. Similar differences have been reported in the eastern white pine-white pine blister rust interaction [22].

Differences in host response to inoculation were first observed at 3 weeks PI, when the fungus began to colonize the developing periderm (phelloderm, phellogen, and phellem) and cortex. At this time point, the rapid development of NP in the resistant clone (DN74) occurred in close proximity to the epidermis [Figure 1], apparently limiting pathogen development in the host. This response is similar to Weiland and Stanosz’s [20] observations of the wound inoculated resistant clone (DN34) at 7 weeks PI. This can be contrasted with differences observed in the susceptible clone (NC11505) between the two studies. Weiland and Stanosz [20] reported the development of multiple successive layers of continuous NP in NC11505 by 7 weeks PI. In this study, the NP in NC11505 did not begin to develop until 5-weeks PI, and was discontinuous, 1 cell layer thick, and inconspicuous by 7 weeks PI, in all sectioned cankers. These results indicate, as suggested by Weiland and Stanosz [20], that NP formation in the susceptible clone was triggered by the wound inoculation protocol. It is interesting to note that there was no visual evidence that the pathogen was able to circumvent NP through disruption by phloem fibers as suggested in the literature [20]. A second explanation may be the differences in terms of the environment (greenhouse vs. field) and the number of cankers per tree between this study and that of Weiland and Stanosz [20]. A third explanation for the differences in NP development in NC11505 is that the pathogen may be able interfere with the defense response of the host. Regardless, the results described above support the idea that the production of NP may be a determining factor in resistance to fungal invasion in the *S. musiva-Populus* interaction.

NP is considered to be part of the restorative process which serves to re-establish the integrity of a tree’s vascular system and the secondary meristem following damage. The response typically begins with the differential expression of genes involved in the phenylpropanoid pathway [32]–[35]. This pathway is essential for lignin biosynthesis and cell wall apposition; necessary steps leading to the development of NP [21]. Mechanical injury and recognition of a pathogen have both been shown to result in activation of the phenylpropanoid pathway [34]. In the case of the pathogen this is typically the result of cell-surface receptors, such as nucleotide-binding site leucine-rich repeat (NBS-LRR) genes, which bind to extracellular ligands produced by the pathogen, triggering a response in the host plant within several minutes to hours of inoculation [23]. The hypersensitive response (HR), a resistance mechanism leading to localized cell death following pathogen recognition [23] is the best characterized of these reactions. HR limits disease development following penetration of biotrophic pathogens by killing a small number of cells in close proximity to the point of infection [23].

In the case of necrotrophic fungi the HR response and localized cell death resulting from recognition of the pathogen leads to susceptibility rather than resistance [23]. Liang *et al.* [36], examining gene expression in hybrid poplar leaves inoculated with *S. musiva* reported an elevated expression of NB-ARC domain-containing disease resistance proteins (NBS-LRR), likely responsible for pathogen recognition, in the susceptible compared to resistant clones. The authors hypothesized that this increased expression may contribute to susceptibility by triggering the necrosis (HR), necessary for pathogen growth. This is consistent with other characterized necrotrophic host-parasite interactions in the literature [32], [34]. However, it is unclear if this model can be applied to understand resistance/susceptibility to stem infection by *S. musiva*, or other forest pathogens.

Anatomical and molecular evidence that fungal pathogens are able to interfere with disease resistance in plants is becoming increasingly common [36]. For example, in the peach-*Leucostoma* interaction the fungus has been shown to alter the structure and location of NP [17]. In a separate study, wounding and inoculation of aspen clones with *Entoleuca mammata* (Wahlenb.) J.D. Rogers & Y.M. Ju (syn. *Hypoxylon mammatum* (Wahl.) Mill.), was demonstrated to delay wound closure regardless of host genotype [37]. This is consistent with the histological responses described above and the results of Weiland and Stanosz [20]. There is also evidence for interference in host resistance at the molecular level. It is thought that this is achieved by altering the hormonal balance in infected plants to control the processes relevant for cell death and accumulation of antimicrobial compounds [37]. The potential interference of host responses in this and other systems by the pathogen requires further investigation. The *S. musiva*-*Populus* interaction is an ideal model to study this phenomenon in trees.

To these authors’ knowledge this is the first study examining canker development in woody tissue without prior wounding. The results from this study indicate that the importance of NP formation in disease resistance may be underestimated, at least in the *S. musiva-Populus* interaction. The lack of significant difference in the early stages of infection between the resistant and susceptible hosts supports the hypothesis that resistance occurs post-penetration [31]. These post-penetration differences are characterized by the lack of NP formation in the susceptible clone compared to the rapid development of NP in the resistant clone. The importance of lenticels and small openings in the infection biology of this fungus suggest that this could be used as a disease resistance screening criteria. However, this would require further examination of variation among clones in terms of lenticel number and structure. These results highlight the importance of using the non-wound inoculation protocol to dissect the *S. musiva-Populus* interaction and develop Septoria canker disease-resistant clones for deployment in the affected regions.

## 
